# Association of early dexamethasone therapy with mortality in critically Ill COVID-19 patients: a French multicenter study

**DOI:** 10.1186/s13613-022-01074-w

**Published:** 2022-10-29

**Authors:** Matthieu Raymond, Aurélie Le Thuaut, Pierre Asfar, Cédric Darreau, Florian Reizine, Gwenhaël Colin, Charly Dano, Julien Lorber, Baptiste Hourmant, Agathe Delbove, Aurélien Frérou, Jean Morin, Pierre Yves Egreteau, Philippe Seguin, Jean Reignier, Jean-Baptiste Lascarrou, Emmanuel Canet

**Affiliations:** 1 Service de Médecine Intensive Réanimation, Centre Hospitalier Universitaire Hôtel-Dieu, 30 Bd. Jean Monnet, 44093 Nantes Cedex 1, France; 2 Direction de la recherche, Plateforme de Méthodologie et Biostatistique, CHU de Nantes, Nantes, France; 3 Service de Médecine Intensive Reanimation, CHU d’Angers, Angers, France; 4 Service de Réanimation Polyvalente, CH du Mans, Le Mans, France; 5 Service de Médecine Intensive Réanimation, CHU de Rennes, Rennes, France; 6 Service de Médecine Intensive Réanimation, CHD de La Roche Sur Yon, La Roche-Sur-Yon, France; 7 Service de Réanimation Polyvalente, CH de Cholet, Cholet, France; 8 Service de Réanimation Polyvalente, CH de Saint Nazaire, Saint-Nazaire, France; 9 Service de Médecine Intensive Réanimation, CHU de Brest, Brest, France; 10 Service de Réanimation Polyvalente, CH de Vannes, Vannes, France; 11 Service de Réanimation Polyvalente, CH de Saint Malo, Saint Malo, France; 12 Unité de Soins Intensifs de Pneumologie, CHU de Nantes, Nantes, France; 13 Service de Réanimation Polyvalente, CH de Morlaix, Morlaix, France; 14 Service de Réanimation Chirurgicale, CHU de Rennes, Rennes, France

**Keywords:** COVID-19, Dexamethasone, Mortality, Intubation, Ventilator-associated pneumonia

## Abstract

**Background:**

Dexamethasone is recommended for COVID-19 patients who require oxygen therapy. However, its effectiveness in reducing mortality and intubation, and its safety, remain debated. We aimed to investigate whether dexamethasone reduces day-28 mortality in unselected patients with critical COVID-19.

**Methods:**

We performed an observational cohort study in consecutive COVID-19 patients admitted to any of 13 French intensive care units (ICUs) in 2020. The primary objective was to determine whether early dexamethasone therapy was associated with day-28 mortality and the secondary objectives were to assess whether early dexamethasone decreased intubation requirements and to collect adverse events.

**Results:**

Of 1058 included patients, 611 (57.75%) received early dexamethasone (early dexamethasone group), 358 (33.83%) did not receive any steroids (no steroids group), and 89 (8.41%) received late dexamethasone or other steroids. Day-28 mortality was similar between the early dexamethasone and the no steroids groups (15.06% and 14.25%, respectively; *P* = 0.59). Factors associated with day-28 mortality were older age (adjusted hazard ratio [aHR], 1.06; 1.04–1.09; *P* < 0.001), worse SOFA score (aHR, 1.13; 1.06–1.20; *P* < 0.001), and immunocompromised status (aHR, 1.59; 1.01–2.50; *P* = 0.043). Early dexamethasone was associated with fewer intubations (48.55% vs. 61.49%, *P* < 0.001) and more ventilator-free days by day 28 (22 [2–28] vs. 17 [1–28] days, *P* = 0.003), compared to no steroids. Ventilator-associated pneumonia (VAP) was more common with early dexamethasone (HR, 1.29 [1.01–1.63], *P* = 0.04) than with no steroids, whereas no differences were noted for bloodstream infection, fungal infection, or gastrointestinal bleeding.

**Conclusions:**

Early dexamethasone in critically ill COVID-19 patients was not associated with lower day-28 mortality. However, early dexamethasone was associated with lower intubation needs and more ventilator-free days by day 28. In patients treated with invasive mechanical ventilation, early dexamethasone was associated with a higher risk of VAP.

**Supplementary Information:**

The online version contains supplementary material available at 10.1186/s13613-022-01074-w.

## Key messages

**What is already known on this topic:**

Dexamethasone decreased day-28 mortality in the randomised controlled trial RECOVERY in patients admitted for COVID-19, including 74% who required oxygen and 16% invasive mechanical ventilation (iMV). Other trials targeting critically ill patients did not replicate this finding, leaving uncertainty about the benefits of dexamethasone.

**What this study adds:**

In our large observational cohort of critically ill COVID-19 patients, of whom 61% required iMV, early systemic dexamethasone was not associated with lower day-28 mortality compared to no steroids. However, early dexamethasone was associated with less need for iMV, more days alive and off iMV, and a higher frequency of ventilator-associated pneumonia in the iMV sub-group.

**How this study might affect research, practice, or policy:**

This study suggests that early dexamethasone may be warranted in critically ill COVID-19 patients, provided those receiving iMV are monitored closely for ventilatory-associated pneumonia.

**Supplementary Information:**

The online version contains supplementary material available at 10.1186/s13613-022-01074-w.

## Background

SARS-CoV-2 is currently responsible for a global pandemic with nearly 500 million known cases and 6 million deaths worldwide as of March 2022 [1]. The disease caused by this coronavirus, COVID-19, can lead to life-threatening complications, of which the most common is acute respiratory distress syndrome (ARDS) [2].

In the randomized controlled RECOVERY trial reported in July 2020, early dexamethasone therapy in a daily dose of 6 mg in patients admitted for COVID-19 decreased day-28 mortality from 25.7–22.9% (*P* < 0.001), compared to usual care. A subgroup analysis of this trial suggested that the benefits of early dexamethasone were greatest in patients who required invasive mechanical ventilation (iMV) [3]. These results were among the main drivers of the decision by the World Health Organization (WHO) to recommend systemic corticosteroid therapy for severely or critically ill patients with COVID-19, in September 2020 [4]. However, RECOVERY did not include patients with medical conditions deemed by the attending physician to put the patient at risk in the event of trial inclusion. Moreover, six other randomized controlled trials found no mortality difference with vs. without corticosteroids in addition to usual care [5–10]. A meta-analysis reported lower mortality with corticosteroid therapy, but this finding was strongly dependent on RECOVERY results and studies evaluating corticosteroid therapy were stopped after the trial publication [11]. In addition, evidence suggests that corticosteroid therapy may increase the risk of ventilator-associated pneumonia (VAP) [12–14], bloodstream infection (BSI) [15], and fungal infections [16, 17].

The primary objective of this retrospective multicenter observational study was to assess day-28 mortality with early dexamethasone therapy (EDT) vs. no steroids in critically ill patients with COVID-19 managed in clinical practice. The secondary objectives were to assess possible differences in intubation needs and adverse events. We hypothesized that EDT would be associated with better survival, as found in the RECOVERY trial.

### Methods

This study was approved by the ethics committee of the French Intensive Care Society (CE SRLF 21-07) on February 11, 2021. In accordance with French law on retrospective studies of anonymized healthcare data, informed consent was not required. This report complies with STROBE guidelines [18].

### Study design, setting, and population

Patients were enrolled between February 1, 2020, and December 31, 2020 at 13 intensive care units (ICUs) in two regions of France (Pays-de-la-Loire and Bretagne, Additional file 1: Appendix S1). Patients were included if they met all of the three following criteria: age ≥ 18 years, positive SARS-CoV-2 polymerase-chain-reaction (PCR) test on a nasopharyngeal swab or respiratory sample, and manifestations of lower respiratory tract infection (fever, dyspnoea, and radiographic lung infiltrates). For patients with multiple admissions during the study period, only the first admission was considered. No patients meeting the inclusion criteria were excluded. Patients admitted between February 1, 2020, and July 1, 2020 were classified as admitted during the first wave of the pandemic, and patients admitted between July 2, 2020, and December 31, 2020 were classified as admitted during the second wave.

### Data collection

For each patient, the data reported in Tables 1, 2, and 3 were extracted from the ICU records and entered by the local investigator at each centre into a standardized web-based electronic case-report form (Castor^®^ Electronic Data Capture System, Amsterdam, The Netherlands; Additional file 1: Appendix S2).

**Table 1:** Baseline characteristics of the study participants

Variables	All patients *N* = 969	Early dexamethasone *N* = 611	No steroids *N* = 358	*P* value
General features				
Age, years, median [IQR]	66.00 [56.00–72.00]	67.00 [59.00–73.00]	65.00 [54.00–71.00]	0.0001
Males, *n* (%)	701 (72.34)	446 (73.00)	255 (71.23)	0.55
Body mass index, kg·m^−2^) ≥ 30, n (%)	397 (41.53)	261 (43.36)	136 (38.42)	0.13
Current smoker, *n* (%)	51 (5.33)	25 (4.14)	26 (7.37)	0.03
Alcohol use, *n* (%)	68 (7.11)	50 (8.28)	18 (5.10)	0.06
Comorbidities				
Diabetes, *n* (%)	289 (29.82)	181 (29.62)	108 (30.17)	0.86
Hypertension, *n* (%)	508 (52.43)	331 (54.17)	177 (49.44)	0.15
Heart disease, *n* (%)	155 (16.00)	109 (17.84)	46 (12.85)	0.04
Underlying immunosuppression, *n* (%)	131 (13.52)	94 (15.38)	37 (10.34)	0.03
Charlson’s index, median [IQR]	3.00 [2.00–5.00]	4.00 [2.00–5.00]	3.00 [1.00–5.00]	< 0.001
Days since COVID-19 symptom onset, median [IQR]	8.00 [6.00–11.00]	8.00 [6.00–11.00]	8.00 [6.00–11.00]	0.90
Location before ICU admission, *n* (%)				
Ward	483 (49.85)	304 (49.75)	179 (50.00)	0.12
Emergency department	429 (44.27)	278 (45.50)	151 (42.18)	
Pre-hospital emergency medical service	57 (5.88)	29 (4.75)	28 (7.82)	

*IQR* interquartile range

**Table 2:** Clinical and laboratory features and ICU management

Variable	All patients *N* = 969	Early dexamethasone *N* = 611	No steroids *N* = 358	*P* value
Clinical parameters at ICU admission				
Respiratory rate, breaths/min, median [IQR]	25.00 [22.00–30.00]	25.00 [21.00–30.00]	25.00 [23.00–30.00]	0.007
Respiratory support at ICU admission				
Standard oxygen, *n* (%)	528 (55.11)	280 (46.05)	248 (70.86)	< 0.001
Noninvasive mechanical ventilation, *n* (%)	14 (1.46)	8 (1.32)	6 (1.71)	
High-flow nasal oxygen, *n* (%)	297 (31.00)	263 (43.26)	34 (9.71)	
Invasive mechanical ventilation, *n* (%)	119 (12.42)	57 (9.38)	62 (17.71)	
Laboratory data				
C-reactive protein, mg/L, median [IQR]	127.70 [73.00–194.00]	122.00 [68.20–188.60]	136.00 [83.10–213.00]	0.07
Fibrinogen, g/L, median [IQR], g/L	6.73 [5.69–7.76]	6.63 [5.57–7.74]	6.90 [6.00–7.91]	0.16
D-dimers, median [IQR]	1147.50 [660.00–2050.00]	1099.00 [652.00–1983.00]	1247.5 [730.00–2472.00]	0.09
SOFA score, median [IQR]	3.00 [2.00–6.00]	3.00 [2.00–4.00]	4.00 [2.00–7.00]	< 0.001
SAPS II, median [IQR]	32.00 [24.00–41.00]	32.00 [24.00–40.00]	33.00 [22.00–44.00]	0.18
Life-sustaining interventions in the ICU, *n* (%)				
Noninvasive mechanical ventilation	82 (8.50)	66 (10.80)	16 (4.52)	< 0.001
High-flow nasal oxygen	552 (57.20)	486 (79.54)	66 (18.64)	< 0.001
Endotracheal mechanical ventilation	571 (58.93)	327 (53.52)	244 (68.16)	< 0.001
ECMO	39 (4.02)	23 (3.76)	16 (4.47)	0.59
Vasopressors	409 (42.30)	230 (37.70)	179 (50.14)	0.0002
Renal replacement therapy	77 (7.95)	42 (6.87)	35 (9.80)	0.10
Other treatments in the ICU, *n* (%)				
Second-line corticosteroids	53 (5.47)	53 (8.67)	–	–
IL6 receptor antagonist	6 (0.62)	6 (0.98)	0 (0.00)	0.09
Convalescent plasma	1 (0.10)	1 (0.16)	0 (0.00)	1.00
Lopinavir ritonavir	79 (8.15)	4 (0.65)	75 (20.95)	< 0.001
Remdesivir	48 (4.95)	37 (6.06)	11 (3.07)	0.04
Other antiviral	8 (0.83)	3 (0.49)	5 (1.40)	0.15
Interferon-β	3 (0.31)	0 (0.00)	3 (0.84)	0.05
Hydroxychloroquine	77 (7.95)	8 (1.31)	69 (19.27)	< 0.001
Ivermectin	22 (2.27)	21 (3.44)	1 (0.28)	0.001
Macrolides	10 (1.03)	4 (0.65)	6 (1.68)	0.19

*ICU* intensive care unit, *IQR* interquartile range, *SOFA* sequential organ failure assessment, *SAPSII* simplified acute physiology score II, *ECMO* extracorporeal membrane oxygenation, *IL6* interleukin 6

**Table 3:** Outcomes

Variable	All patients *N* = 969	Early dexamethasone *N* = 611	No steroids *N* = 358	*P* value
Day-28 mortality (primary outcome), *n* (%)	143 (14.8)	92 (15.1)	51 (14.3)	0.58^a^
Secondary outcomes				
Adverse events in the ICU, *n* (%)				
Bloodstream infection	87 (9.0)	58 (9.5)	29 (8.1)	0.19^b^
Ventilator-associated pneumonia	277 (48.5)	173 (52.9)	104 (42.6)	0.04^b^
Invasive fungal infection	43 (4.4)	26 (4.3)	17 (4.8)	0.72^c^
Gastrointestinal bleeding	32 (3.3)	24 (3.9)	8 (2.2)	0.16^c^
Intubation in the ICU, *n* (%)	450/848 (53.1)	268/552 (48.6)	182/296 (61.49)	< 0.001^b^
Time from ICU admission to iMV, days, median [IQR]	1.00 [0.00–2.00]	1.00 [0.00–2.00]	0.00 [0.00–1.00]	< 0.001^d^
iMV-free days on day 28, median [IQR]	20.0 [1.0–28.0]	22.0 [2.0–28.0]	17.0 [1.0–28.0]	< 0.003^d^
Renal replacement therapy, *n* (%)	77 (8.0)	42 (6.9)	35 (9.8)	0.03^b^
Alive and out of the ICU on day 28, *n* (%)	652 (67.3)	410 (67.1)	242 (67.3)	0.89^e^
90-day mortality *n* (%)	188 (19.4)	126 (20.6)	62 (17.3)	0.16^a^

*ICU* intensive care unit, *iMV* invasive mechanical ventilation, *IQR* interquartile range

^a^Frailty model

^b^Fine and Gray model for competing risks

^c^Logistic regression

^d^Student’s test

^e^chi-square test

Patients were classified in the early dexamethasone group if they received intravenous dexamethasone before or within 48 h after ICU admission and in the no steroids group if they did not receive any steroids during the ICU stay. Patients who received late dexamethasone (after 48 h of ICU admission) or other steroids where reported separately but excluded from the analyses. An exploratory analysis has been conducted where patients who received other steroids before or within 48 h after ICU admission where included in the early dexamethasone group.

### Objectives

The primary study objective was to compare day-28 mortality between the early dexamethasone and no steroids groups. The secondary objectives were to assess the proportions of patients who required invasive mechanical ventilation (iMV), the number of ventilator-free days by day 28, and adverse events categorised as infections (VAP, bloodstream infection [BSI], and invasive fungal infection) or gastro-intestinal bleeding.

## Statistical analysis

Variables were described for the overall population and for each of the two groups. We computed *n* (%) for qualitative data and used the chi-square test or Fisher's exact test, as appropriate, to compare the groups. Quantitative data were described as mean ± SD if normally distributed and as median [interquartile range] otherwise; comparisons were with Student's *t* test and the Mann–Whitney test, respectively.

Kaplan–Meier plots of survival in the two groups were compared using the log-rank test.

To identify factors associated with day-28 mortality, we started by performing a univariate shared-frailty Cox analysis to take centre into account as a random effect. All variables associated with *P* values  < 0.20 were then included in a multivariate model. Time from the onset of COVID-19 symptoms and pandemic wave were included in the model because of their clinical importance. A descending step-by-step procedure was used to keep only variables associated with *P* values  < 0.05. Sub-group analyses predefined according to the current knowledge from the literature were performed in an effort to identify populations characterised by differences in variables associated with EDT. Furthermore, to address nonrandomized treatment allocation, we calculated propensity scores by multivariable logistic regression with early course of dexamethasone as the binary outcome and age, SOFA score, Charlson’s index, Respiratory rate, Respiratory support at ICU admission, underlying immunosuppression, and days since onset of symptoms as covariates. Using such propensity scores, we applied inverse probability of treatment weighting (IPTW) to create a pseudo-study cohort, where the weighted version can balance off the covariate bias and mimic a randomized treatment assignment situation: the IPT weights for dexamethasone-treated patients = 1/p (treated); for untreated patients = 1/(1− p [treated]) [19].

The proportions of intubated patients in the two groups were compared using the Fine-and-Gray method [19], with death as the competing event. Confounding factors were sought, and a multivariate model was built. The Fine-and-Gray method was also applied to compare the proportions of patients with VAP, BSI, and invasive fungal infection, with death as the competing event. Confounding factors were also sought and a multivariate model built. Each infection category was then analysed separately. Finally, the number of iMV-free days by day 28 was compared between groups using the Wilcoxon Mann–Whitney test.

## Results

**Fig. 1:**
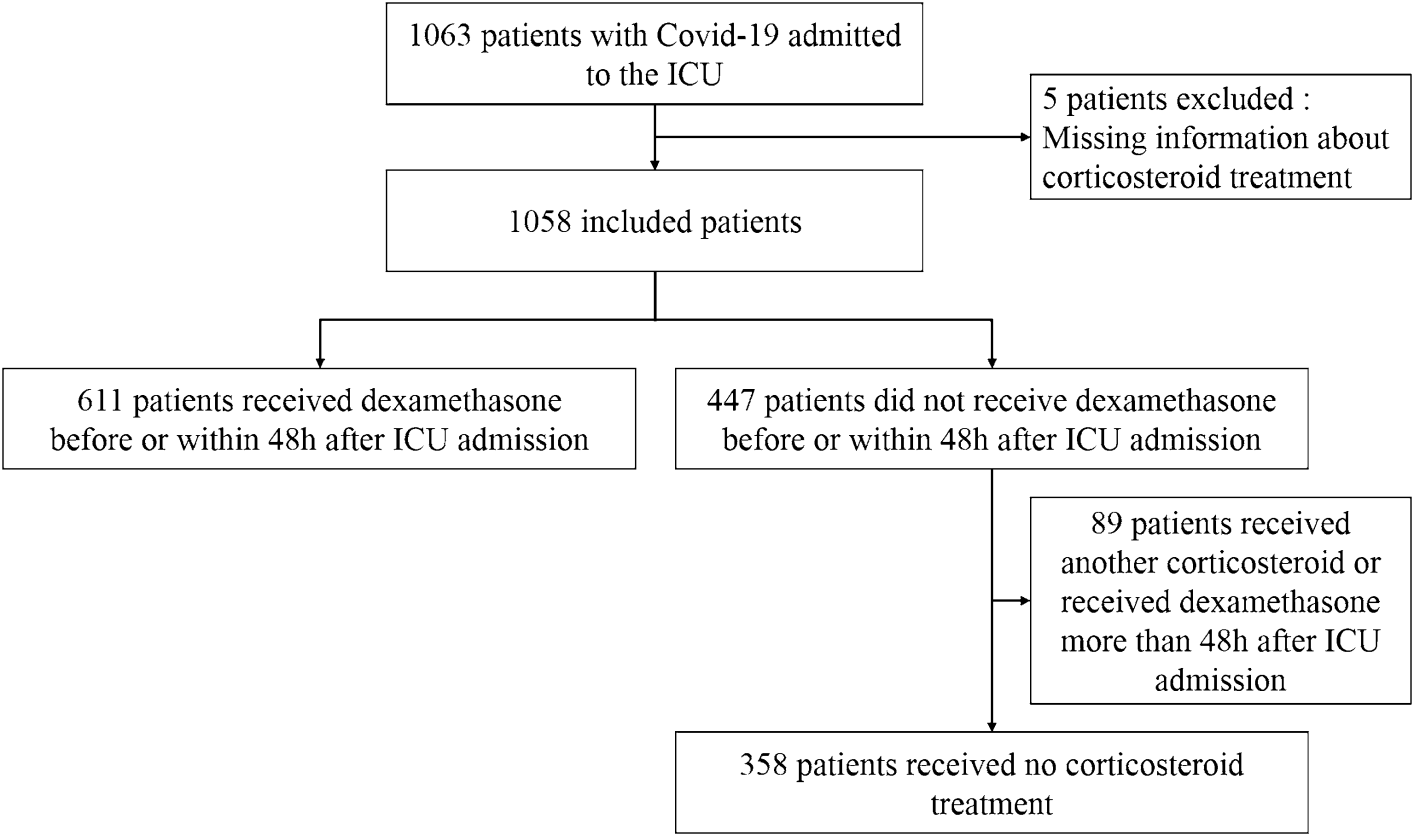
Patient flowchart

Figure 1 shows the flowchart. Of the 1058 patients, 611 received EDT, 358 did not receive any corticosteroid therapy, and 89 received late dexamethasone or other steroids during the ICU stay.

### Study population

Table 1 reports the main features of the patients. All patients had acute respiratory failure responsible for severe hypoxemia with a mean PaO_2_/FiO_2_ ratio of 199 ± 93 mmHg at ICU admission. Of the 969 patients, 571 required iMV.

### Details of corticosteroid treatment

In the early dexamethasone group, the median prednisone-equivalent daily dosage was 40 [40–40] mg and the median treatment duration was 9 [9–10] days.

Among the 89 patients who received late dexamethasone or other corticosteroids, 44 (49.4%) received methylprednisolone, 20 (22.5%) hydrocortisone, 15 (16.9%) prednisolone, and 10 (11.2%) late dexamethasone. The mean prednisone-equivalent daily dosage was 134 ± 188 mg, initiated 6 ± 10 days after ICU admission, and the mean treatment duration was 9 ± 9 days. Among these 89 patients, 48 had the treatment initiated after 48 h of ICU admission (methylprednisolone *n* = 24, hydrocortisone *n* = 12, dexamethasone *n* = 10, prednisolone *n* = 2), and 41 had the treatment initiated before or within 48 h after ICU admission (methylprednisolone *n* = 20, prednisolone *n* = 13, hydrocortisone *n* = 8).

### Primary outcome

**Fig. 2:**
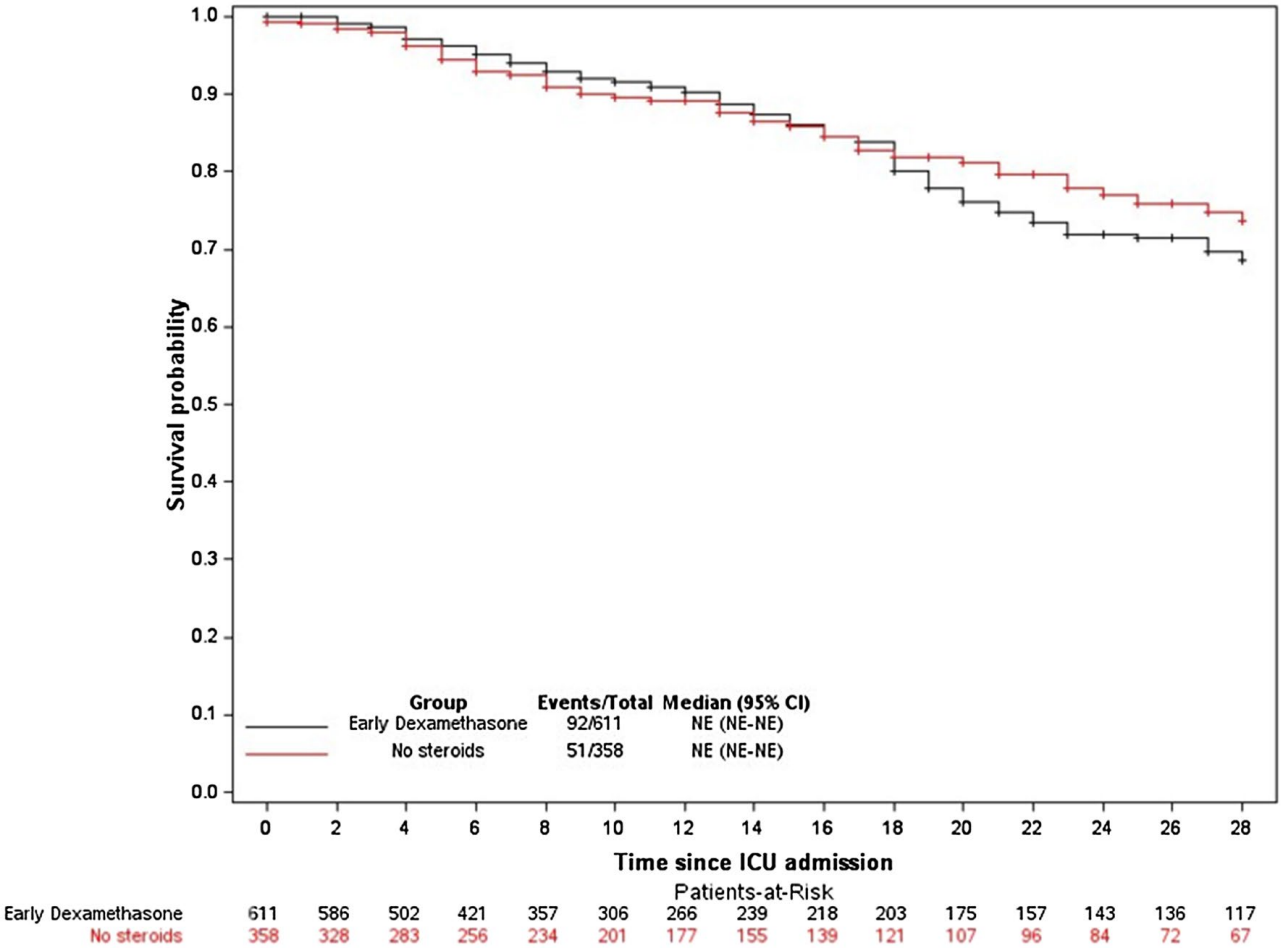
Day-28 mortality in the groups with early dexamethasone therapy vs. without any steroids. Survival curves were compared using Cox regression

**Fig. 3:**
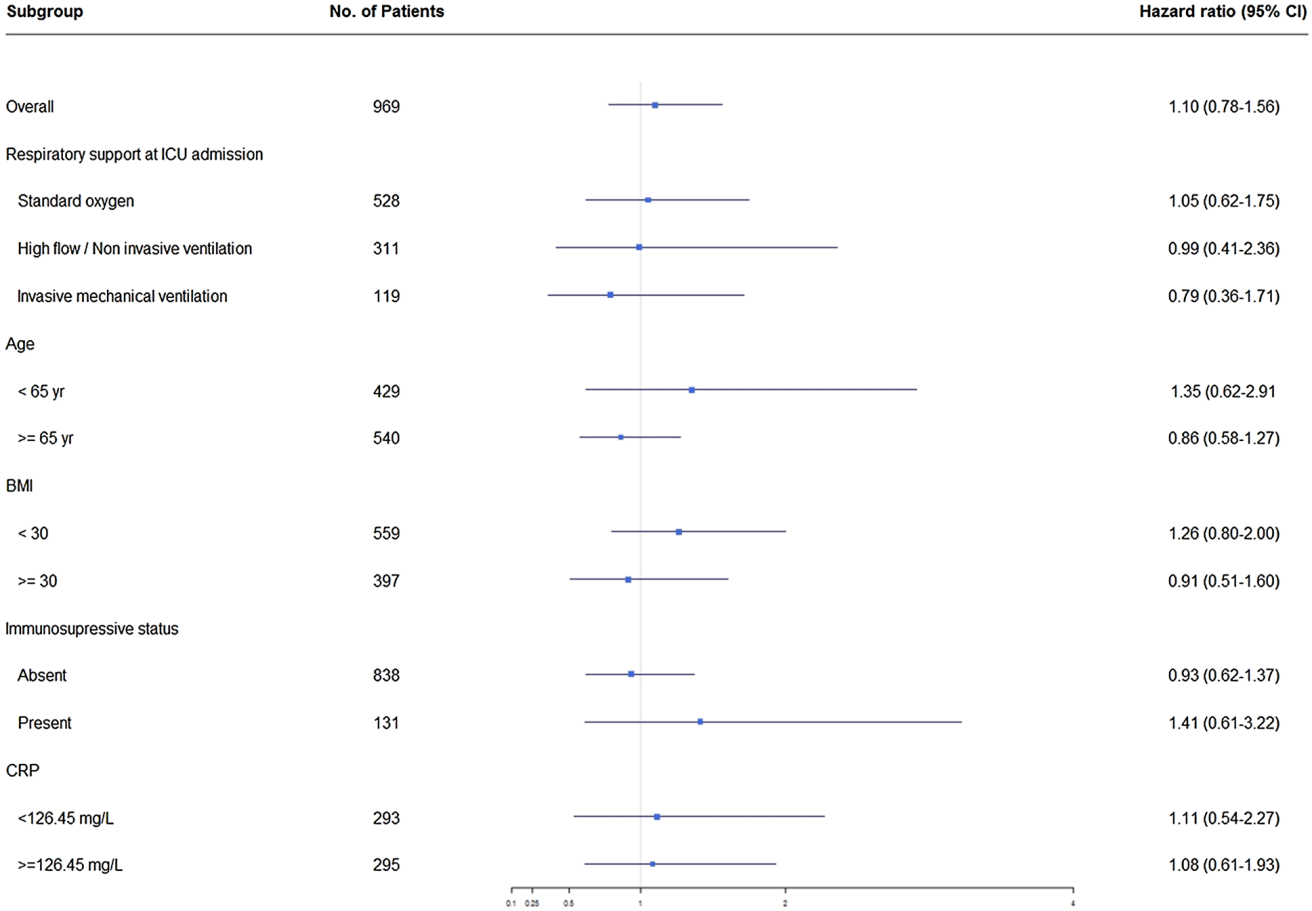
Forest plot of the subgroup analysis for day-28 mortality. *BMI* body mass index, *CRP* C-reactive protein, *ICU* intensive care unit

Tables 2 and 3 report details on management and outcomes, respectively. Day-28 mortality was not significantly different between the two groups (Fig. 2). By multivariable analysis, factors associated with higher day-28 mortality were older age, underlying immunosuppression, and worse SOFA score on ICU day 1; EDT was not associated with day-28 mortality in the overall population (Additional file 1: Table S1). An exploratory analysis comparing the early steroids group (where the 41 patients who received other steroids before or within 48 h following ICU admission were added to the early dexamethasone group) to the late steroids group (48 patients who received dexamethasone or other steroids after 48 h of ICU admission) and to the group of patients who did not receive any steroids produced similar results (Additional file 1: Tables S1, S2). Day-28 mortality was also similar in all prespecified subgroups (Fig. 3). Finally, using propensity score analysis, we found no association between the EDT and day-28 mortality (hazard ratio [HR], 1.15; 95% confidence interval [95% CI] 0.90–1.46; *P* = 0.27).

### Secondary outcomes

**Fig. 4:**
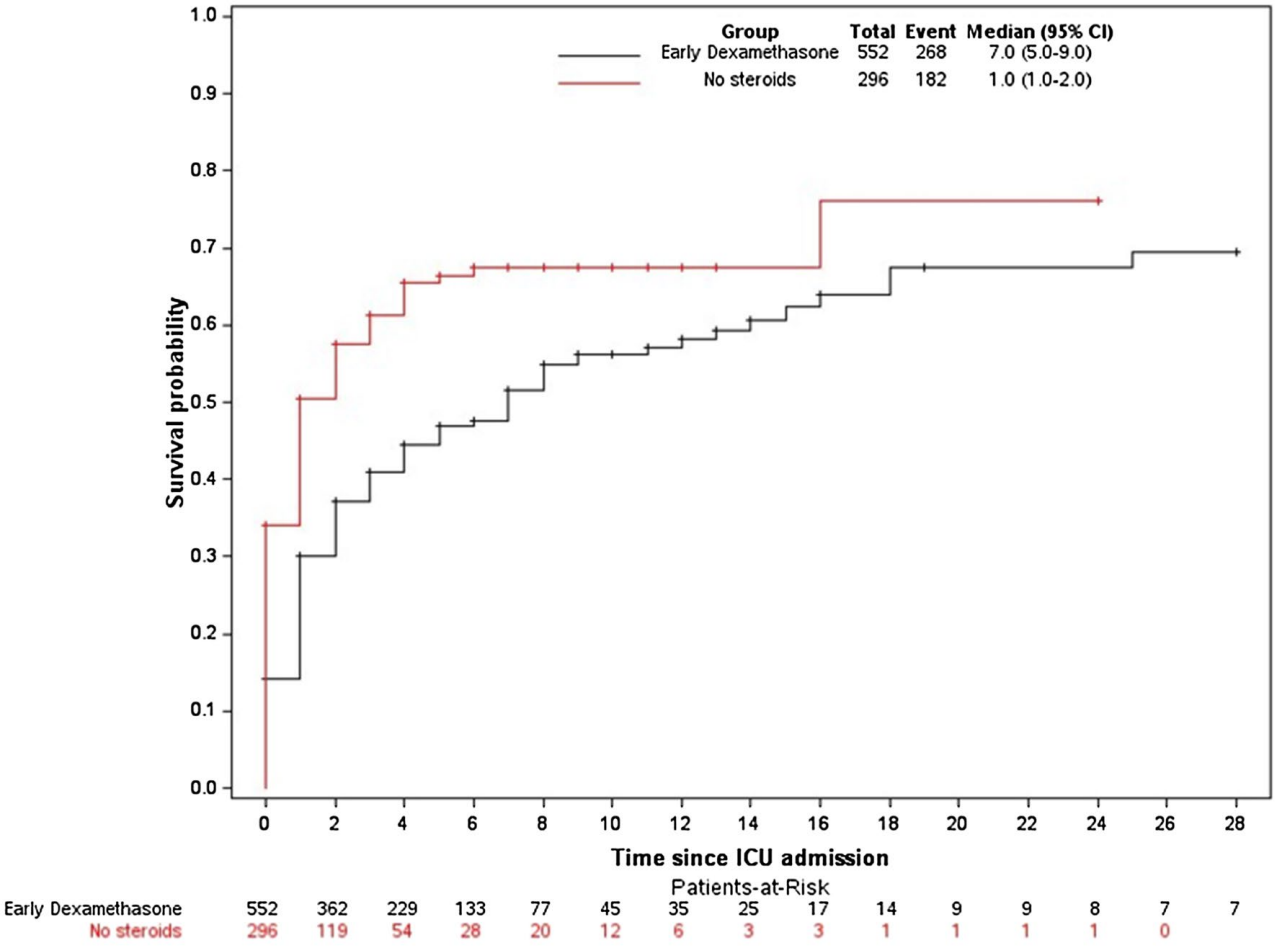
Probability of intubation during the ICU stay according to treatment group

The fine-and-gray analysis with death as the competing event showed that EDT was significantly associated with a lower risk of iMV (HR, 0.60; 95%CI 0.50–0.71; *P* < 0.0001) (Fig. 4). This difference was also found by multivariate analysis adjusted for respiratory rate, PaO_2_/FiO_2_ ratio, and respiratory support at ICU admission (adjusted HR, 0.49; 95% CI 0.40–0.59; *P* < 0.0001) (Additional file 1: Table S4). Moreover, the number of ventilator-free days by day 28 was higher in the EDT group than in the no steroids group (22 [2–28] vs. 17 [1–28], *P* < 0.003).

VAP was more common in the EDT group than in the no steroids group (HR, 1.29; 1.01–1.63; *P* = 0.04) (Additional file 1: Fig. S1). In contrast, the frequencies of BSI, invasive fungal infections, and gastrointestinal bleeding were similar in the two groups (Table 3 and Additional file 1: Fig. S2).

## Discussion

In this large multicenter cohort study of critically ill COVID-19 patients, EDT was not associated with lower day-28 mortality compared to no steroids. However, the EDT group had a lower proportion of patients who required iMV and a higher number of ventilator-free days by day 28, compared to the no steroids group. Finally, in the subgroup of patients treated with iMV, VAP was more common with vs. without EDT.

ARDS is associated with high risks of death [20] and long-term morbidities [21]. The recommendation by experts to give systemic corticosteroid therapy to patients with ARDS rests on randomised controlled trials of only moderate quality and remains controversial [22] Moreover, corticosteroid therapy might delay viral clearance, increase viral dissemination, and raise the risk of nosocomial infections [23]. Serious adverse events have been inconsistently reported in previous studies [11]. Our EDT group had a higher frequency of VAP compared to the usual-care group. This finding underlines the possibility of harm from corticosteroid therapy in some patients.

The RECOVERY trial demonstrated a reduction in day-28 mortality with vs. without dexamethasone therapy [3]. Whereas our cohort included only critically ill patients, among whom 61% required iMV, only 16% of patients in the RECOVERY trial needed iMV and 24% needed no supplemental oxygen at all. However, RECOVERY showed a trend toward greater benefits of dexamethasone in the patient sub-group on iMV. On the other hand, subsequent trials in patients with greater disease severity compared to those included in RECOVERY also found no decrease in day-28 mortality with corticosteroid therapy [5–10]. In RECOVERY, that 15% of recruited patients were considered ineligible for dexamethasone therapy may have introduced bias. In addition, in patients receiving respiratory assistance in the usual-care groups, day-28 mortality in RECOVERY was almost twice that in our study (29% vs. 14.3%). Our EDT group experienced some benefits, consisting of a decreased need for iMV, and a higher number of ventilator-free days. Overall, these data suggest that the effects of dexamethasone therapy may vary with disease severity, with the greatest benefits occurring in patients in the middle of the spectrum. Moreover, recent studies reported that the effects of corticosteroid treatment on mortality may vary according to some patients’ characteristics with greater benefits in older patients with a pro-inflammatory phenotype and worse outcomes in those with a hypoinflammatory phenotype of COVID-19 ARDS [24–26]. Further research is needed to investigate whether the administration of dexamethasone could individualized in critically ill COVID-19 patients.

The limitations of our study include the observational design, which precludes the assessment of a potentially causal relationship between EDT and patient outcomes. Second, the patients were enrolled over 11 months in 2020, during which changes in respiratory-support strategies, with greater use of HFNO and NIV for COVID-19 patients occurred. However, the type of respiratory support was included in the multivariable model designed to identify factors associated with iMV. Moreover, there was no centre effect. Third, the diagnosis of VAP was not confirmed by an adjudication committee, and the frequency of VAP may, therefore, have been overestimated. Each local investigator used the criteria recommended in Europe [27]. In addition, the overestimation would have occurred similarly in the two groups. Fourth, decisions of treatment limitations (do not intubate or do no resuscitate orders) were not recorded in our study and could have influenced the analysis on factors associated with intubation. Nonetheless, such decisions would have occurred in the two groups and are unlikely to modify our findings. A major strength of our study is that it provides information on everyday clinical practice. Thus, our study included patients who would not have been eligible for randomised trials. Our population was large, with very few missing data, and was recruited at multiple centres. In contrast to many other studies, we looked for associations between EDT and the need for iMV. Finally, we carefully recorded adverse effects of dexamethasone.

## Conclusions

Our findings support EDT as a component of standard care for critically ill COVID-19 patients. Although day-28 mortality was not lower with EDT, the lower frequency of iMV and greater number of ventilator-free days were important benefits expected to improve patient comfort and preserve ICU resources. Nonetheless, the higher frequency of VAP seen with EDT indicates a need for close monitoring of patients on iMV. Conceivably, EDT may benefit the most to patients not treated with iMV, with disease severity in the intermediate range.

## Supplementary Information

**Additional file 1: Appendix S1.** List of the 13 participating intensive care units in France. **Appendix S2.** Supplementary Methods. **Table S1.** Multivariable analysis to identify factors associated with day-28 mortality. **Table S2.** Univariate analysis to identify factors associated with day-28 mortality among the 3 groups of patients (Early steroids, late steroids, and no steroids). **e-Table S3.** Multivariable analysis to identify factors associated with day-28 mortality among the 3 groups of patients (Early steroids, late steroids, and no steroids). **Table S4.** Multivariable analysis to identify factors associated with intubation. **Figure S1.** Ventilator-associated pneumonia: probability in each treatment group. **Figure S2.** Bloodstream infection: probability in each treatment group.

## Data Availability

The data sets used and/or analysed during the current study are available from the corresponding author on reasonable request.

## Abbreviations

aHR: Adjusted hazard ratio
ARDS: Acute respiratory distress syndrome
BMI: Body mass index
BSI: Bloodstream infection
CRP: C-reactive protein
ECMO: Extracorporeal membrane oxygenation
eCRF: Electronic case report form
EDT: Early dexamethasone therapy
FiO_2_: Fraction of inspired oxygen
HFNO: High-flow nasal oxygen
HR: Hazard ratio
ICU: Intensive care unit
iMV: Invasive mechanical ventilation
IQR: Interquartile range
NIV: Noninvasive ventilation
PaO_2_: Arterial oxygen partial pressure
PCR: Polymerase chain reaction
SAPSII: Simplified acute physiology score version II
SOFA: Sequential organ failure assessment
VAP: Ventilator-associated pneumonia

## References

[1] COVID-19 Map—Johns Hopkins Coronavirus Resource Center. https://coronavirus.jhu.edu/map.html. Accessed 14 Jan 2021

[2] COVID-ICU Group on behalf of the REVA Network and the COVID-ICU Investigators (2021). Clinical characteristics and day-90 outcomes of 4244 critically ill adults with COVID-19: a prospective cohort study. Intensive Care Med.

[3] RECOVERY Collaborative Group, Horby P, Lim WS, Emberson JR, Mafham M, Bell JL, et al. Dexamethasone in hospitalized patients with Covid-19. N Engl J Med. 2021;384(8):693–704. 10.1056/NEJMoa2021436. Epub 2020 Jul 17. 10.1056/NEJMoa2021436 PMC7383595 32678530

[4] Corticosteroids for COVID-19. https://www.who.int/publications-detail-redirect/WHO-2019-nCoV-Corticosteroids-2020.1. Accessed 14 Jan 2021

[5] Villar, J. Efficacy of dexamethasone treatment for patients With ARDS Caused by COVID-19. clinicaltrials.gov 2021 Feb. Report No: NCT04325061. https://clinicaltrials.gov/ct2/show/NCT04325061

[6] Tomazini BM, Maia IS, Cavalcanti AB, Berwanger O, Rosa RG, Veiga VC (2020). Effect of dexamethasone on days alive and ventilator-free in patients with moderate or severe acute respiratory distress syndrome and COVID-19: the CoDEX randomized clinical trial. JAMA.

[7] Dequin P-F, Heming N, Meziani F, Plantefève G, Voiriot G, Badié J (2020). Effect of hydrocortisone on 21-day mortality or respiratory support among critically Ill patients with COVID-19: a randomized clinical trial. JAMA.

[8] Scandinavian Critical Care Trials Group. Low-dose Hydrocortisone in patients With COVID-19 and severe hypoxia—the COVID steroid trial. clinicaltrials.gov 2021 Sep. Report No: NCT04348305. https://clinicaltrials.gov/ct2/show/NCT04348305

[9] Derde L. Randomized, embedded, multifactorial adaptive platform trial for community-acquired Pneumonia. clinicaltrials.gov; 2021 Dec. Report No. NCT02735707. https://clinicaltrials.gov/ct2/show/NCT02735707

[10] Peking Union Medical College Hospital. Glucocorticoid therapy for critically ill patients with severe acute respiratory infections caused by COVID-19: a prospective, randomized controlled trial. clinicaltrials.gov. Report No. NCT04244591. https://clinicaltrials.gov/ct2/show/NCT04244591. 2020

[11] Sterne JAC, Murthy S, Diaz JV, Slutsky AS, Villar J, WHO Rapid Evidence Appraisal for COVID-19 Therapies (REACT) Working Group (2020). Association between administration of systemic corticosteroids and mortality among critically Ill patients with COVID-19: a meta-analysis. JAMA.

[12] Blonz G, Kouatchet A, Chudeau N, Pontis E, Lorber J, Lemeur A (2021). Epidemiology and microbiology of ventilator-associated pneumonia in COVID-19 patients: a multicenter retrospective study in 188 patients in an un-inundated French region. Crit Care Lond Engl.

[13] Razazi K, Arrestier R, Haudebourg AF, Benelli B, Carteaux G, Decousser J-W (2020). Risks of ventilator-associated pneumonia and invasive pulmonary aspergillosis in patients with viral acute respiratory distress syndrome related or not to Coronavirus 19 disease. Crit Care Lond Engl.

[14] Maes M, Higginson E, Pereira-Dias J, Curran MD, Parmar S, Khokhar F (2021). Ventilator-associated pneumonia in critically ill patients with COVID-19. Crit Care Lond Engl.

[15] Buetti N, Ruckly S, de Montmollin E, Reignier J, Terzi N, Cohen Y (2021). COVID-19 increased the risk of ICU-acquired bloodstream infections: a case-cohort study from the multicentric OUTCOMEREA network. Intensive Care Med.

[16] Machado M, Valerio M, Álvarez-Uría A, Olmedo M, Veintimilla C, Padilla B (2021). Invasive pulmonary aspergillosis in the COVID-19 era: an expected new entity. Mycoses.

[17] Koehler P, Bassetti M, Chakrabarti A, Chen SCA, Colombo AL, Hoenigl M (2021). Defining and managing COVID-19-associated pulmonary aspergillosis: the 2020 ECMM/ISHAM consensus criteria for research and clinical guidance. Lancet Infect Dis.

[18] von Elm E, Altman DG, Egger M, Pocock SJ, Gøtzsche PC, Vandenbroucke JP (2007). The strengthening the reporting of observational studies in epidemiology (STROBE) statement: guidelines for reporting observational studies. Lancet Lond Engl.

[19] Austin PC, Stuart EA (2015). Moving towards best practice when using inverse probability of treatment weighting (IPTW) using the propensity score to estimate causal treatment effects in observational studies. Stat Med.

[20] Bellani G, Laffey JG, Pham T, Fan E, Brochard L, Esteban A (2016). Epidemiology, patterns of care, and mortality for patients with acute respiratory distress syndrome in intensive care units in 50 countries. JAMA.

[21] Angus DC, Clermont G, Linde-Zwirble WT, Musthafa AA, Dremsizov TT, Lidicker J (2006). Healthcare costs and long-term outcomes after acute respiratory distress syndrome: a phase III trial of inhaled nitric oxide. Crit Care Med.

[22] Annane D, Pastores SM, Rochwerg B, Arlt W, Balk RA, Beishuizen A (2017). Guidelines for the diagnosis and management of critical illness-related corticosteroid insufficiency (CIRCI) in critically Ill patients (part I): society of critical care medicine (sccm) and European society of intensive care medicine (ESICM) 2017. Crit Care Med.

[23] Moreno G, Rodríguez A, Reyes LF, Gomez J, Sole-Violan J, Díaz E (2018). Corticosteroid treatment in critically ill patients with severe influenza pneumonia: a propensity score matching study. Intensive Care Med.

[24] Amado-Rodríguez L, Salgado Del Riego E, Gomez de Ona J, López Alonso I, Gil-Pena H, López-Martínez C (2022). Effects of IFIH1 rs1990760 variants on systemic inflammation and outcome in critically ill COVID-19 patients in an observational translational study. J eLife.

[25] Sinha P, Furfaro D, Cummings MJ, Abrams D, Delucchi K, Maddali MV (2021). Latent class analysis reveals COVID-19-related acute respiratory distress syndrome subgroups with differential responses to corticosteroids. Am J Respir Crit Care Med.

[26] Torres A, Motos A, Cillóniz C, Ceccato A, Fernández-Barat L, Gabarrús A (2022). Major candidate variables to guide personalised treatment with steroids in critically ill patients with COVID-19: CIBERESUCICOVID study. Intensive Care Med.

[27] Surveillance of healthcare-associated infections and prevention indicators in European intensive care units: HAI-Net ICU protocol, version 2.2 Eur Cent Dis Prev Control. 2017 https://www.ecdc.europa.eu/en/publications-data/surveillance-healthcare-associated-infections-and-prevention-indicators-european. Accessed 26 May 2022

