# Supplementary material for: Association of early dexamethasone therapy with mortality in critically Ill COVID-19 patients: a French multicenter study
Source: Ann Intensive Care. 2022 Oct 29;12:102. doi: 10.1186/s13613-022-01074-w (PMC9617242; doi:10.1186/s13613-022-01074-w)
Supplement: Supplementary file 1 — Additional file 1: Appendix S1. List of the 13 participating intensive care units in France. Appendix S2. Supplementary Methods. Table S1. Multivariable analysis to identify factors associated with day-28 mortality. Table S2. Univariate analysis to identify factors associated with day-28 mortality among the 3 groups of patients (Early steroids, late steroids, and no steroids). e-Table S3. Multivariable analysis to identify factors associated with day-28 mortality among the 3 groups of patients (Early steroids, late steroids, and no steroids). Table S4. Multivariable analysis to identify factors associated with intubation. Figure S1. Ventilator-associated pneumonia: probability in each treatment group. Figure S2. Bloodstream infection: probability in each treatment group. [file 13613_2022_1074_MOESM1_ESM.docx]

**SUPPLEMENTAL MATERIAL**

**Contents**

e-Appendix 1: List of participating centres…………………………………………. p 2

e-Appendix 2: Supplementary Methods…………………………………………….. p 3

e-Table 1: Multivariable analysis to identify factors associated with day-28 mortality.. p 6

e-Table 2: Univariate analysis to identify factors associated with day-28 mortality among the 3 groups of patients (Early steroids, late steroids, and no steroids) ………………………p 7

e-Table 3: Multivariable analysis to identify factors associated with day-28 mortality among the 3 groups of patients (Early steroids, late steroids, and no steroids)….……….……….p 8

e-Table 4: Multivariable analysis to identify factors associated with intubation……. p 9

e-Figure 1: Ventilator-associated pneumonia: probability in each treatment group…. p 10

e-Figure 2: Bloodstream infection: probability in each treatment group………..…… p 11

**e-Appendix 1: List of the 13 participating intensive care units in France**

| **List of the participating centres** | **Number of patients included N=969** | **Early dexamethasone N=611** | **Mortality**  **N (%)** | **No steroids N=358** | **Mortality**  **N (%)** |
| --- | --- | --- | --- | --- | --- |
| Intensive Care Unit, University Hospital, Nantes | 150 (15.48%) | 105 (17.18%) | 14 (13.3%) | 45 (12.57%) | 11 (24.4%) |
| Intensive Care Unit, University Hospital, Angers | 166 (17.13%) | 91 (14.89%) | 24 (26.4%) | 75 (20.95%) | 12 (16.0%) |
| Intensive Care Unit, Community Hospital, Le Mans | 131 (13.52%) | 85 (13.91%) | 5 (5.9%) | 46 (12.85%) | 6 (13.0%) |
| Intensive Care Unit, University Hospital, Rennes | 108 (11.15%) | 52 (8.51%) | 4 (7.7%) | 56 (15.64%) | 3 (5.4%) |
| Intensive Care Unit, Regional Hospital Centre, La Roche sur Yon | 103 (10.63%) | 74 (12.11%) | 9 (12.2%) | 29 (8.10%) | 4 (13.8%) |
| Intensive Care Unit, Community Hospital, Cholet | 64 (6.60%) | 49 (8.02%) | 11 (22.4%) | 15 (4.19%) | 3 (20.0%) |
| Intensive Care Unit, Community Hospital, Saint Nazaire | 56 (5.78%) | 39 (6.38%) | 10 (25.6%) | 17 (4.75%) | 0 (0%) |
| Intensive Care Unit, University Hospital, Brest | 51 (5.26%) | 32 (5.24%) | 3 (9.4%) | 19 (5.31%) | 1 (5.3%) |
| Intensive Care Unit, Community Hospital, Vannes | 53 (5.47%) | 19 (3.11%) | 2 (10.5%) | 34 (9.50%) | 8 (23.5%) |
| Intensive Care Unit, Community Hospital, Saint Malo | 32 (3.30%) | 19 (3.11%) | 4 (21.0%) | 13 (3.63%) | 3 (23.1%) |
| Pulmonary Intensive Care Unit, University Hospital, Nantes | 21 (2.17%) | 20 (3.27%) | 4 (20.0%) | 1 (0.28%) | 0 (0%) |
| Intensive Care Unit, Community Hospital Morlaix | 19 (1.96%) | 16 (2.62%) | 0 (0%) | 3 (0.84%) | 0 (0%) |
| Surgical Intensive Care Unit, University Hospital, Rennes | 15 (1.55%) | 10 (1.64%) | 2 (20.0%) | 5 (1.40%) | 0 (0%) |

**e-Appendix 2: Supplementary Methods**

***Data collection***

For each patient, the data were extracted retrospectively from the ICU records and entered by the local investigator into an electronic case report form ((Castor^®^ Electronic Data Capture System, Amsterdam, The Netherlands). The collected data were age and sex, comorbidities and Charlson Comorbidity Index,^1^ SARS-CoV-2 infection characteristics, bacterial co-infection if present, date of ICU admission, baseline characteristics and severity (SOFA score^2^ and SAPSII score^3^) at ICU admission, treatments in the ICU (corticosteroid therapy, antiviral drugs, immunomodulators, antibiotics, anticoagulation, and vasopressors), changes in oxygen supplementation and ventilatory assistance during the ICU stay, acute kidney injury and need for renal replacement therapy, nosocomial infections (ventilator-associated pneumonia [VAP], bloodstream infection [BSI], and invasive fungal infection, with microbiological documentation and treatment), thrombotic complications, bleeding events, ICU stay length, day-28 mortality, and day-90 mortality. For corticosteroid therapy, the type, dosage, and duration were also recorded. Pharmacological therapy was not standardized among centers. Prescriptions of antiviral drugs and immunomodulators were left at the discretion of the intensivist in charge or administered because some patients were included in clinical trials.

**Definitions**

Patients were classified as immunocompromised if they had any of the following conditions: solid-organ transplantation, human immunodeficiency virus infection, haematopoietic stem-cell transplantation, haematological malignancy, solid malignancy (new diagnosis or current progression or in remission for less than five years), corticosteroid treatment before COVID-19 onset and for longer than 30 days, treatment with another immunosuppressive drug, or known primary immunodeficiency.

VAP was defined as hospital-acquired pneumonia diagnosed after at least 48 h of invasive mechanical ventilation (iMV) or within 48 hours after extubation. The diagnosis of VAP was established by the intensivist in charge of the patient using criteria recommended by the European Centre for Disease Prevention and Control:^4^ new radiological lung infiltrates combined with at least one systemic sign (temperature >38.3°C not due to another cause and/or leukocyte count <4000/mm^3^ or >12000/mm^3^) and with one or more respiratory signs (new onset of purulent sputum or change in character of sputum and/or worsening gas exchange plus at least one positive microbiological sample [quantitative cultures of a distal blind protected-specimen brush, with a threshold of 10^3^ colony-forming units/mL; or of a bronchoalveolar-lavage specimen, with a threshold of 10^4^ colony-forming units/mL; or of an endotracheal aspirate, with a threshold of 10^6^ colony-forming units/mL; or positive culture of a pleural-fluid specimen).

BSI was defined as at least one blood sample drawn more than 48 h after ICU admission and positive for bacteria or fungi. For coagulase-negative staphylococci and other common skin contaminants, at least two consecutive blood cultures positive for the same pathogen at different times and sites were required.

Fungal infections were defined as infections meeting AspICU criteria for invasive aspergillosis.^5^

We defined ventilator-free days as the total number of calendar days or portions of calendar days of unassisted breathing during the first 28 days after randomization. All the patients who had died by day 28 were considered to have had no ventilator-free days. ^6^

**References**

1. Brusselaers N, Lagergren J. The Charlson Comorbidity Index in Registry-based Research. Methods Inf Med. 2017;56(5):401‑6.

2. Vincent JL, Moreno R, Takala J, Willatts S, De Mendonça A, Bruining H, et al. The SOFA (Sepsis-related Organ Failure Assessment) score to describe organ dysfunction/failure. On behalf of the Working Group on Sepsis-Related Problems of the European Society of Intensive Care Medicine. Intensive Care Med. juill 1996;22(7):707‑10.

3. Le Gall JR, Lemeshow S, Saulnier F. A new Simplified Acute Physiology Score (SAPS II) based on a European/North American multicenter study. JAMA. 22 déc 1993;270(24):2957‑63.

4. Surveillance of healthcare-associated infections and prevention indicators in European intensive care units: HAI-Net ICU protocol, version 2.2 [Internet]. European Centre for Disease Prevention and Control. 2017 [cité 26 mai 2022]. Disponible sur: https://www.ecdc.europa.eu/en/publications-data/surveillance-healthcare-associated-infections-and-prevention-indicators-european

5. Blot SI, Taccone FS, Van den Abeele AM, Bulpa P, Meersseman W, Brusselaers N, et al. A clinical algorithm to diagnose invasive pulmonary aspergillosis in critically ill patients. Am J Respir Crit Care Med. 1 juill 2012;186(1):56‑64.

6 Schoenfeld DA, Bernard GR. Statistical evaluation of ventilator-free days as an efficacy measure in clinical trials of treatments for acute respiratory distress syndrome. Crit Care Med 2002;30:1772-1777.

**e-Table 1: Multivariable analysis to identify factors associated with day-28 mortality**

| **Variable** | **HR** | **95%CI** | ***P* value** |
| --- | --- | --- | --- |
| Early dexamethasone therapy | 0.43 | 0.13–1.46 | 0.177 |
| Immunocompromised status | 1.59 | 1.01–2.50 | 0.043 |
| Age^a^ | 1.06 | 1.04–1.09 | <0.001 |
| Charlson’s Comorbidity Index^b^ | 1.07 | 0.99–1.16 | 0.079 |
| SOFA score on day 1^b^ | 1.13 | 1.06–1.20 | <0.001 |
| Time from the onset of first symptoms ^c^ | 1.00 | 0.96-1.05 | 0.882 |
| Admission during the second pandemic wave | 1.75 | 0.51-6.01 | 0.371 |

HR: hazard ratio; 95%CI: 95% confidence interval; SOFA: Sequential Organ Failure Assessment

^a^HR for each 1-year increase

^b^HR for each 1-point increase

^c^HR for each 1-day increase

**e-Table 2: Univariate analysis to identify factors associated with day-28 mortality among the 3 groups of patients (Early steroids, late steroids, and no steroids)**

Univariate shared-frailty Cox analysis to take centre into account as a random effect.

| **Variable** | **HR** | **95%CI** | ***P* value** |
| --- | --- | --- | --- |
| No steroids | 1 |  | 0.867 |
| Early steroids ^a^ | 1.09 | 0.78–1.54 |  |
| Late steroids ^b^ | 1.02 | 0.54–1.91 |  |

^a^ Early steroids : 652 patients who received steroids before or within 48 hours following ICU admission (Dexamethasone n=611, methylprednisolone n=20, prednisolone n=13, hydrocortisone n=8).

^b^ Late steroids : 48 patients who received steroids after 48 hours of ICU admission (Methylprednisolone n=24, hydrocortisone n=12, prednisolone n=2).

**e-Table 3: Multivariable analysis to identify factors associated with day-28 mortality among the 3 groups of patients (Early steroids, late steroids, and no steroids).**

| **Variable** | **HR** | **95%CI** | ***P* value** |
| --- | --- | --- | --- |
| No steroids | 1 |  | 0.251 |
| Early steroids | 0.72 | 0.49–1.06 |  |
| Late steroids | 0.83 | 0.42–1.62 |  |
| Immunocompromised status | 1.70 | 1.11–2.61 | 0.015 |
| Age^a^ | 1.06 | 1.03–1.08 | <0.001 |
| Charlson’s Comorbidity Index^b^ | 1.07 | 0.99–1.15 | 0.068 |
| SOFA score on day 1^b^ | 1.15 | 1.08–1.21 | <0.001 |
| Time from the onset of first symptoms ^c^ | 1.02 | 0.98-1.06 | 0.241 |

HR: hazard ratio; 95%CI: 95% confidence interval; SOFA: Sequential Organ Failure Assessment

^a^HR for each 1-year increase

^b^HR for each 1-point increase

^c^HR for each 1-day increase

**e-Table 4: Multivariable analysis to identify factors associated with intubation**

| **Variable** | **HR** | **95%CI** | ***P* value** |
| --- | --- | --- | --- |
| Early dexamethasone treatment | 0.49 | 0.40–0.59 | <0.001 |
| Respiratory rate at admission^a^ | 1.03 | 1.01–1.04 | <0.001 |
| PaO_2_/FiO_2_ at ICU admission^b^ | 0.94 | 0.92–0.95 | <0.001 |
| HFNO or NIV at admission (ref.: standard oxygen) | 0.86 | 0.69–1.07 | 0.17 |

HR: hazard ratio; 95%CI: 95% confidence interval; HFNO: high-flow nasal oxygen; ICU: Intensive Care Unit; NIV: noninvasive ventilation

^a^HR for each additional breath/minute

^b^HR for each 10-point increase

**e-Figure 1: Ventilator-associated pneumonia: probability in each treatment group**

**
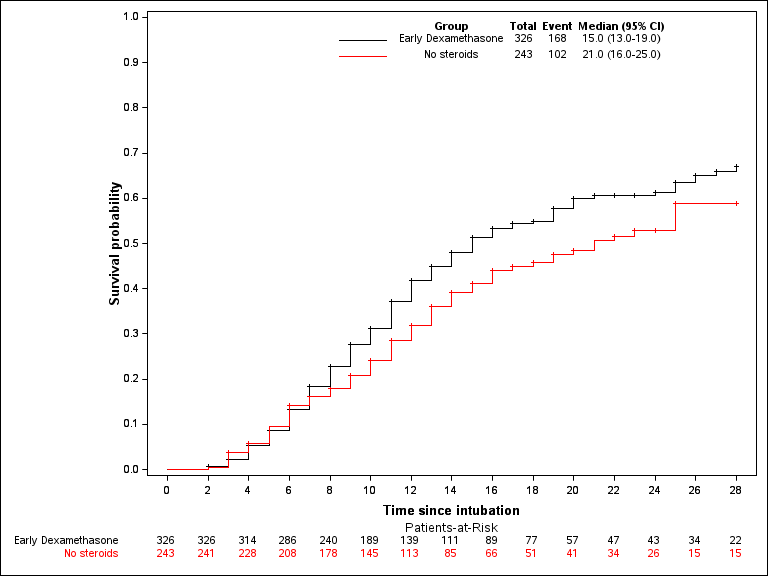
**

**e-Figure 2: Bloodstream infection: probability in each treatment group**

**
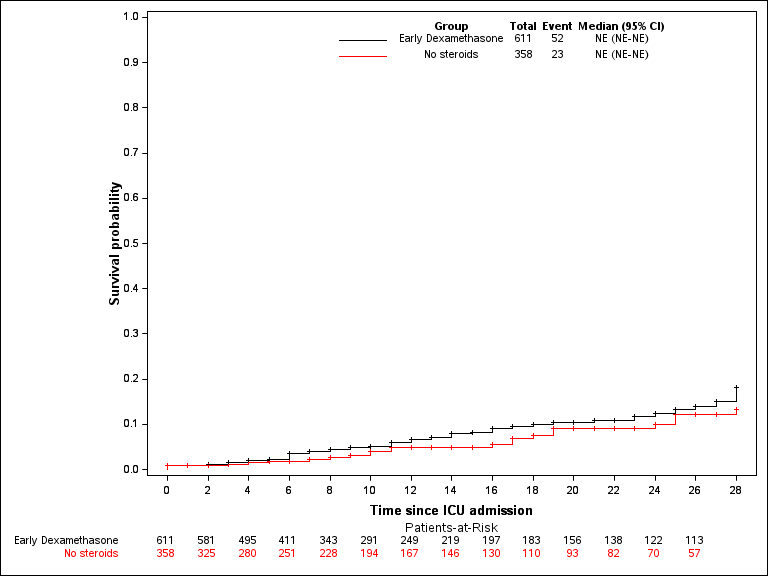
**
